# Development of a method and an assessment construct for person-centered translation of dementia public stigma scales

**DOI:** 10.3389/fpubh.2023.1233400

**Published:** 2024-01-23

**Authors:** Junfeng Lu, Yi Shan, Meng Ji, Lee-Fay Low, Sarang Kim, Annica Barcenilla-Wong, Sam Shen, Weiwei Chu

**Affiliations:** ^1^College of International Studies, Jiaxing University, Jiaxing, China; ^2^School of Languages and Cultures, University of Sydney, Sydney, NSW, Australia; ^3^Faculty of Medicine and Health, University of Sydney, Sydney, NSW, Australia; ^4^Australian Institute of Health and Welfare (AIHW), Canberra, ACT, Australia; ^5^College of Foreign Languages and Literature, Fudan University, Shanghai, China

**Keywords:** development, method, assessment, construct, person-centered, translation, dementia, stigma

## Abstract

**Background:**

With the number of people with dementia dramatically increasing over time and dementia becoming a major health concern worldwide, scales have been developed to assess the stigma socially attached to this neurodegenerative disorder. There are, however, almost no available methods and assessment constructs for person-centered translation of dementia public stigma scales.

**Objective:**

To develop such a method and such an assessment construct by translating the Dementia Public Stigma Scale (DPSS) into standard written Chinese.

**Methods:**

We translated the DPSS following three major steps: (1) literal translation and mistranslation identification; (2) panel discussions of items with problematic translations; and (3) the final checking of the translated scale. Informed by the translation and adaptation process, we then developed a method for person-centered translation of dementia public stigma scales. Based on this method and our panel discussions, we finally proposed a tripartite assessment construct for quality evaluation of the translation of dementia public stigma scales.

**Results:**

Forward and backward translation did not work sufficiently in dementia public stigma scale translation. Mistranslations were induced by three major causes, including confusion caused by multiple Chinese meanings of the immediate Chinese direct translation, the lack of immediate Chinese direct translation because of varying positive/negative emotions attached to multiple translations, and the lack of culture-specific idioms in Chinese. Based on these factors, we proposed a tripartite dementia translation assessment construct. Following this assessment tool, we determined the best Chinese version that could further be tested for its psychometric properties among the public.

**Conclusion:**

A method and an assessment construct for person-centered translation of dementia public stigma scales were developed. Such a method and such an assessment construct could be followed in the translation of dementia public stigma scales and the translation evaluation of such scales.

## Introduction

### Prevalence of dementia and dementia-related stigma

With the number of people with dementia dramatically increasing over time (1), dementia is regarded as a major health concern worldwide (2). About 50 million individuals are currently diagnosed with dementia globally and without a medical breakthrough, this is projected to rise to 131.5 million by 2050 (3). Of this amount, an apparently increasing proportion will be identified in Latin America, Africa, India, China, South Asia, and the Western Pacific region (4), due to some reasons, including health and care systems often providing limited or no support to people with dementia or their families in these low-and middle-income countries and regions, much higher increasing proportions of older people in low-and middle-income countries compared with that in higher-income countries, etc. (3). The number of people living with dementia in China has been estimated to be 9.5 million in the population aged 60 years or older (1). Despite the high prevalence and growing trend of dementia in China, this neurodegenerative disorder is conceptually stigmatized in contemporary Chinese society (5). In the Chinese context of cultural, social, and political undesirabilities characterizing such a disorder, it is increasingly stigmatized in China (5). The increased public awareness that the mind constitutes a key concern in maintaining a high quality of life in contemporary China reinforces the persistence of dementia-related stigma in the public, which manifests itself in the form of silencing, indifference, or ignorance in memory clinics or other public settings (5). In this background of research, it is imperative to provide a scale assessing dementia public stigma in China to deliver targeted education and interventions and launch dementia stigma reduction initiatives.

Growing evidence has shown that dementia is regarded as one of the most feared health conditions (6). Some people with dementia experience social stigmas (7) caused by fear and the lack of public awareness and understanding of dementia (8). These stigmas include dementia-related stereotypes, negative prejudices and emotional reactions, and discriminatory behaviors (9). Dementia-related stigmas bring about a potential barrier to care and support (10, 11) that can manifest itself in such behaviors as excluding individuals with dementia in healthcare decisions (12) or shunning family members of individuals living with dementia (13). However, there is limited research focusing on dementia stigma and few evidence-based interventions specifically targeting dementia stigma (14), although reducing dementia stigma can contribute to better care access, greater support engagement, and ultimately higher life quality for individuals with dementia and their families (7).

### Stigma as a social construct

Stigma is a perspective “generated in social contexts” (15), where a socially salient group difference is identified, devalued, and used as a source of discrimination against individuals or groups (16). Stigma consists mainly of public stigma, affiliated stigma, and self-stigma (16). Both public and self-stigma include three components: stereotypes, prejudice, and discrimination. Public stigma consists of negative beliefs about a group, agreement with belief and/or negative emotional reaction, and behavioral response to prejudice (16). Public stigmatizing views are not limited to uninformed members of the general public, and even well-trained professionals from most mental health disciplines subscribe to stereotypes about mental illness (16). Self-stigma comprises negative beliefs about the self, agreement with beliefs and negative emotional reactions, and behavioral responses to prejudice (16). Affiliated stigma has been shown to limit the social support and social opportunities available to family members who come to share some of the shame, blame, and loss associated with their family members’ stigma(s) (17). As observed by Jones and Corrigan, public stigma underpins affiliated stigma and self-stigma (18). Based on this observation, we believe that it is imperative to study public stigma before examining affiliated and self-stigmas.

Stigma has been widely viewed as a social construct in the literature. Goffman regards stigma as “spoiled identity,” a gap between “virtual social identity” (how a person is characterized by society) and “actual social identity” (the attributes possessed by a person) (15). As such, the stigmatizing process is relational: the social environment defines what is deviant and provides the context where devaluing evaluations are expressed (19). According to the Modified Labeling Theory, stigma is a social construct in which powerful groups in society impose negative stereotypical labels on those who are deemed undesirable and subsequently devalued and subjected to discrimination (20). Crocker et al. (21) also define stigma socially. They claim that stigma occurs when a person is believed to possess an “often objective” characteristic conveying a particular devalued social identity in a specific social context (21). Such an identity is socially constructed by defining who belongs to a specific social group and whether an attribute will lead to a given devalued social identity in a particular social context (22). Like Goffman (15), Crocker et al. (21) define stigmas as an essentially “devaluing social identity” that occurs within a particular social context that defines a feature as devaluing. Since stigma is socially constructed and dependent on relationship and context (23), the sociocultural environment where stigma occurs (20) and the myriad societal forces that shape exclusion from social life (24) need to be considered in stigma-related studies. Considering the sophistication of stigma as a complex social construct, we think it advisable to explore public stigma before investigating affiliated and self-stigma when it comes to dementia.

### Developing socioculturally-relevant dementia public stigma scales

#### The relevance of the worldwide study and translation of dementia public stigma

Despite the high prevalence of 131.5 million individuals living with dementia worldwide by 2050 (2), negative attitudes toward and discrimination against people with dementia are quite common (25, 26). Dementia-related stigmas bring about wide-ranging consequences, such as low self-esteem, poor psychological well-being, social isolation, and poor quality of life (9). It is, therefore, imperative to develop psychometrically sound scales to measure dementia knowledge and dementia-related stigma. The Dementia Knowledge Assessment Scale has been developed to support dementia knowledge evaluation in diverse populations and inform educational intervention development, and it has been proven valid and reliable for assessing knowledge deficiencies and change in those caring for and treating people with dementia (27). Such instruments are essential for providing knowledge about how to develop interventions for dementia-related stigma reduction in the community (9).

Some dementia stigma scales have been developed to assess such stigma. Stigma Questionnaire (28), STIG-MA (29), and Dementia Stigma Questionnaire (30) were adapted from multiple sources. However, these instruments have been rarely adopted till now (9). The validated Family Stigma in Alzheimer’s disease Scale reflects caregiver stigma, lay public stigma, and structural stigma (31). It was designed to assess family members’ perceptions of the stigma held by the public rather than lay public attitudes toward people living with Alzheimer’s disease (9). The validated Dementia Attitudes Scale (32) assesses people’s positive attitudes to people with dementia rather than common stereotypes or negative attitudes toward dementia and people with dementia (9). It is also not designed to measure structural discrimination or perceived personhood (e.g., enjoying life and interaction) that might be regarded as an essential aspect of dementia stigma underlying and impacting individual stigmatizing attitudes and behaviors (33). Originally developed and validated as a tool assessing perceived stigma against HIV/AIDS and cancer (34), the Stigma Impact Scale was revised to measure stigma perceived by persons with dementia and their caregivers (35). Although it has been proven effective among its target respondents, it was not designed to assess dementia public stigma. The Perceived Psychiatric Stigma Scale (36) was suitable and effective for measuring perceived public stigma in Chinese social and cultural settings, but it is designed to measure stigma attached to mental illnesses rather than to the neurodegenerative disorder of dementia. To better capture dementia public stigma, stereotypes of people with dementia, such as being dangerous (37), being a burden to family and the health care system, being incapable of speaking for themselves, being unreliable, and being unable to contribute to the society (38), need to be covered in dementia public stigma scales.

#### Established methods for health survey language translation

There are many approaches to health survey language translation methodology (39), including forward translation, back-translation, team-based translation, pretest of the translated scales, etc. Among these approaches, back translation is regarded as the most common persisting methodology used to translate mental health materials (40). Back-translation prioritizes equivalence between the source and target texts (41). However, this approach cannot truly ensure equivalence. A translation may be assumed equivalent when the back-translated text is not equivalent to the source text because of problematic translation that may not be identified during the translation process (41), especially when many mental health-related terms are particularly challenging or even impossible to translate directly (40).

Another widely adopted approach is the TRAPD (translation, review, adjudication, pretesting, and documentation) model (42, 43). Although there is no consensus on research standards to evaluate the quality of a translation, the TRAPD is considered the gold standard for questionnaire translation and adaptation. This model advocates a team-based approach through which a team of researchers (translators) with diverse expertise jointly produce an optimal version of the tool, as translation skills alone are not sufficient in a survey context (44). The TRAPD focuses on cultural equivalence rather than on word-or entity-level literal equivalence (45). Due to its general design purpose, this translation approach is not perfectly applicable to the translation of dementia public stigma scales because it is not sufficiently person-centered. Drawing on the team-based approach proposed by the TRAPD, we took a little step forward by fully considering the personhood of people with dementia in our team-based translation process in this study.

#### Developing a method and an assessment construct for the translation of dementia public stigma scales by translating the DPSS into Chinese

Herrmann et al. (7) reviewed worldwide evidence on dementia stigma over the past decade, focusing on how stigmatizing attitudes may present themselves in various ethnic subgroups, stigma assessment instruments, and prospective or experimental approaches to stigma assessment and management. As they discovered, only one cross-sectional study was conducted by Cheng et al. in China (7). Cheng et al. (28) found lower levels of stigma in participants with relatives or friends living with dementia and in younger and more educated individuals using 11 English assessment items derived from other stigma scales (34, 46–48). The assessment tool of Cheng et al. developed through synthesizing diverse currently available evaluation instruments may, to some extent, be neither sufficiently systematic in assessment nor adequately relevant to the target sociocultural context. A scale appropriate to the Chinese language and culture is needed to assess dementia public stigma among Chinese populations. Currently, there is no available dementia public stigma scale developed in the Chinese language to adopt targeted approaches to countering or eliminating dementia-related stigma, including protest, education, and contact (49). In this context, translating already-developed tools for use is a rapid and practical approach to assessment (50) before delivering more tailored stigma-mitigating interventions or launching more targeted stigma-reducing initiatives.

Given painstaking efforts as well as considerable time and cost investments involved in developing new instruments (50) and the purpose of establishing international comparability across different studies, well-developed, available, and reliable instruments need to be adapted and validated cross-linguistically (51, 52). As such, there is a pressing need to translate quantitative scales into the language of the culture in which these tools are adopted (53). In the development of well-established and scientifically validated instruments available in various languages, scientific standards must be meticulously followed during translation, adaptation, and comprehensive psychometric evaluation. To this end, strategies need to be used in the whole translation and adaptation process to ensure semantic equivalence and cultural appropriateness, including “forward translation, semantics evaluation and consolidation of the translated version, back translation, translation equivalence testing, and further adaptation” (54). Based primarily on the forward-backward translation approach, these strategies are designed to adapt an instrument in “a culturally relevant and comprehensible form” without changing its original meaning and intent (55). Such strategies are informative and helpful for the translation of the original English version of the DPSS into Chinese, but they are probably not sufficient in such a translation mainly for two reasons. The first reason is concerned with the different lexical systems, different language registers, and distinct cultural expression repertoires between the source and target languages and cultures. The second factor relates to cultural differences (56) in both perceived and experienced stigma (10) between Chinese and English cultural settings. To ensure a successful translation of this scale and help develop a dementia public stigma reduction initiative (7) in China, we aimed to develop a person-centered translation method that could produce culturally acceptable dementia public stigma scales by achieving semantic closeness and accuracy and cultural relevance and to develop an assessment construct for evaluating the translation of dementia public stigma scales. In the context that stigmatizing attitudes can be displayed in various ethnic subgroups, stigma assessment instruments, and prospective or experimental approaches to stigma assessment and management worldwide (7), the method and the assessment construct for person-centered translation of dementia public stigma scales we proposed in this study could directly help reduce dementia public stigma that presents itself in stigma assessment scales and stigma assessment and management approaches.

Our development of such a translation method was inspired by Kitwood (57), who attaches great importance to the “personhood” of people with dementia and defines it as “a standing or status that is bestowed upon one human being by others in the context of relationship and social being” (57). However, malignant social psychology undermines the personhood of individuals with dementia (57). As such, Kitwood (57) proposes person-centered care and underpins good dementia care within relationships, interconnectedness, and communication between people, by postulating that nurses need to serve as role models to enable family and the public who contact the person with dementia to replicate person-centered practices. Our proposal and development of a method and an assessment construct for person-centered translation of dementia public stigma scales can somehow contribute to the reduction of malignant social psychology or socially attached stigma toward the person with dementia and to the popularization of person-centered dementia care.

## Design and methods

### Overall design

This study was conducted at Jiaxing University, China, and the University of Sydney, Australia from February 1 to May 8, 2023. First, we translated and adapted the DPSS following three major steps. Informed by the translation and adaptation process, we then developed a method for person-centered translation of dementia public stigma scales. Based on this method and our panel discussions during translation and adaptation, we finally proposed a three-item assessment construct for the quality evaluation of the translation of dementia public stigma scales.

### The dementia public stigma scale

To address the need for assessing dementia-related public stigma, Kim et al. (9) drew on the Attribution Theory to develop and validate the DPSS that comprises the three components of the tripartite model of stigma (cognitive, emotional, and behavioral) (58, 59). Social-psychologically oriented, the Attribution Theory proposes that public stigma comprises three components: stereotypes, prejudice, and discrimination (58). Within the framework of this theory, stereotypes refer to generalized negative beliefs about a specific group, prejudice means the negative emotional reactions to these stereotypes, and discrimination is a negative behavioral reaction caused by prejudice (58). Based on the Attribution Theory (58), the DPSS can facilitate understanding the formative factors underpinning stigma and allow for a more nuanced exploration of dementia stigma and its impacts across or within populations. To our knowledge, the DPSS is the latest and most systematic scale for assessing dementia public stigma.

The DPSS is a five-factor, 16-item construct. The five factors are Fear and Discomfort (Items 1–4), Incapability (Items 5–9), Personhood (Items 10–12), Burden (Items 13–14), and Exclusion (Items 15–16). Responses to the 16 items are measured through a seven-point Likert scale ranging from 1 (strongly disagree) to 7 (strongly agree). The total scores achievable for this tool, therefore, vary from 16 to 112. Six items are reverse-scored (1, 2, 3, 10, 11, and 12). As regard the other items, a higher score indicates a more negative attitude toward dementia. The DPSS displayed moderate to high reliability in all five factors (Cronbach’s α = 0.805 for Factor 1, 0.738 for Factor 2, 0.743 for Factor 3, 0.796 for Factor 4, and 0.743 for Factor 5). The whole scale also showed high reliability (Cronbach’s α = 0.818). Item analysis also indicated that removing any of the 16 items would not increase Cronbach’s Alpha value. Capturing the cognitive, emotional, and behavioral domains of stigma, Kim et al. (9) have effectively validated the factor structure of the DPSS that underpins dementia pubic stigma among their study participants (9).

As found by Kim et al. (9), the DPSS is a comprehensive, valid, and reliable tool among community-dwelling adults in the Australian sociocultural context, which can not only be used to measure the public stigma of dementia among adults but also be used to develop and evaluate interventions for dementia-related stigma reduction. However, the DPSS may not be completely applicable to other sociocultural contexts, considering that there is no accepted “gold standard” for assessing dementia-related stigma (7) as stigma is a complex social construct shaped by the sociocultural environment (20) and various social forces (24). As such, it is relevant to translate and adapt the DPSS and other systematic scales, if any, to diverse languages and cultures and study dementia public stigma in these linguistic-cultural contexts for intervention purposes. The translated and adapted scales then need to be validated through psychometric evaluation to test their validity and reliability. However, such psychometric evaluation is out of the purview of the current study and will be conducted in future studies.

### Developing the Chinese version of the DPSS

Based on our analysis of the studies reported by Herrmann et al. (7), particularly Cheng et al. (28), the dementia-related expertise of four authors (L-FL, SK, AB-W, and SS) of our study, and our consultations with some mental health professionals working at the Hospital Affiliated with Jiaxing University and Qilu Hospital of Shandong University, we believed that the brief, user-friendly, and quick-to-complete assessment instrument of the DPSS could reveal dementia public stigma in the Chinese sociocultural context if well translated and adapted to the Chinese language and culture.

Drawing on and developing the methodologies adopted in previous studies (53–55, 60–62), we developed the Chinese version of the DPSS following three major steps below.

1. First, three translators (Meng Ji, Yi Shan, and Weiwei Chu) translated the DPSS into Chinese.
2. A panel comprising bilingual health educators, bilingual translators, the scale author, and content experts met to discuss items with problematic translations and corresponding root causes by double-checking the target version against the source version. Discussion of the meaning of the items and possible translations was undertaken until consensus was obtained. An adapted English item was sometimes written in conjunction with the Chinese translation. The consequences of forced literal translation and their implications for translation were also worked out through panel discussion.
3. The final translated scale was sent back to all panel members for checking.

### Developing a method and an assessment construct for person-centered translation of dementia public stigma scales

The development of a method and an assessment construct for person-centered translation of dementia public stigma scales was informed conceptually by the translation and adaptation guidelines reported in relevant studies (53–55, 60–62) and practically by the accumulated health translation experience of three authors of this study (MJ, YS, and WC) and the translation process above. Thus informed, we focused on panel discussions after literal translation, making full use of the potential advantages of the panel members: the language proficiency of native Chinese speakers (MJ, YS, and WC) and native English speakers (L-FL, SK, AB-W, and SS); the health translation experience of bilingual translators (MJ, YS, and WC); and the expertise of the scale author of the DPSS (SK) and content experts (L-FL, AB-W, and SS) who are engaging in studies on mental health with a special focus on dementia. Such penal discussions ensured not only the linguistic appropriateness and comprehensibility as well as cultural relevance and accessibility of the translated scale but also the maintenance of the original meaning and intent of the source scale (54). The method developed was presented schematically in the RESULTS section. Based on this method and our panel discussions, we finally proposed a three-item assessment construct for the quality evaluation of the translations of dementia public stigma scales, which was also provided in the RESULTS section.

Kim (63) proposed a people-centered theory of translation by advocating a focus on “what people need, what people can do, and what people think and feel” in translation. Informed by this proposal and Kitwood’s (57) advocacy of person-centered care, we tentatively developed a method of person-centered translation of dementia public stigma scales by mainly considering the dignity and self-esteem of persons with dementia and showing understanding of and sympathy for them from multiple perspectives of the health translators, the DPSS author, and dementia experts who well understand persons with dementia. We were thus concerned with upholding the personhood of people with dementia and catering to linguacultural appropriateness and relevance in the Chinese sociocultural context while maintaining the original meaning and intent of the DPSS when we addressed mistranslations and agreed upon the final Chinese version of the DPSS. We also put forth three items of evaluation from the perspective of persons with dementia when proposing the assessment construct. Overall, such a person-centered orientation was implemented throughout the entire process of our study.

### Ethical considerations

This study was approved by the Academic Committee of the College of International Studies, Jiaxing University, China. We conducted this research among all authors of this paper without involving study informants.

## Results

**Figure 1:**
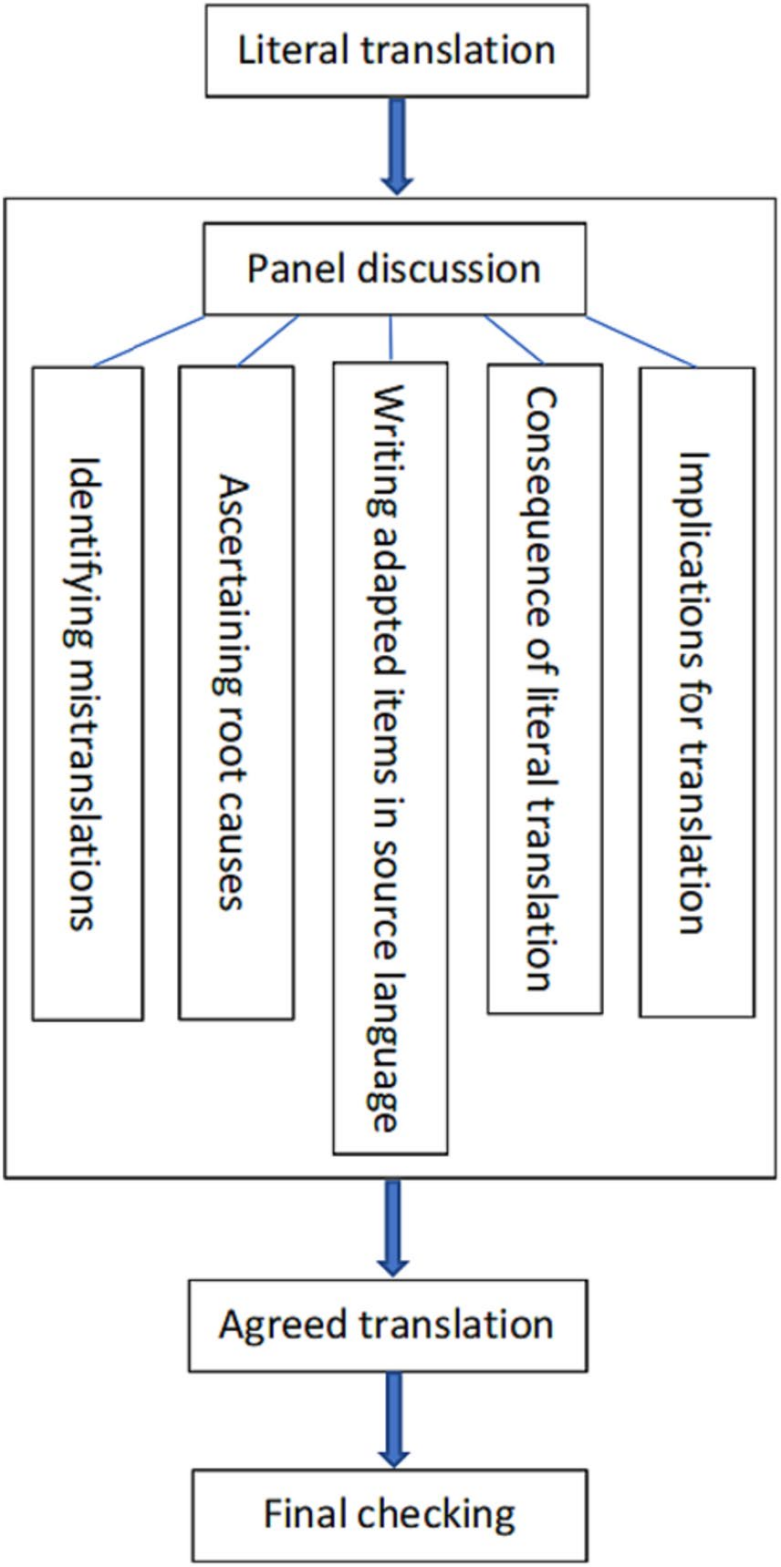
Method for person-centered translation of dementia public stigma scales.

The method for the person-centered translation of dementia public stigma scales we developed could be displayed schematically in Figure 1. We conducted six rounds of translation (see Table 1 in the *Identification of the best translation among* var*ious translation options* subsection below), each of which followed the processes described in Figure 1, to produce the best-translated version of the DPSS. In the development of such a method, we (1) used inclusive, non-offensive words that were friendly to people with dementia and their families, (2) centered on the person rather than on the neurodegenerative disorder or the social care system by considering “what people need, what people can do and what people think and feel” (63), and (3) focused on practice and cognition to enhance translatability (63). The following subsections of this section illustrate with examples how we produced the person-centered version of the DPSS to avoid stripping individuals of their dignity and self-esteem, reinforcing inaccurate stereotypes, and heightening the fear and stigma surrounding dementia (64). We will revisit the concept of “person-centered translation” and how to achieve it in detail in the section “Discussion.”

**Table 1:** Dementia public stigma scale translation assessment.

Translation variants	Semantic meaning closeness to the English words (SMC)	Perceived cultural familiarity/acceptability to target readers (PCF)	Perceived psychological harm to target readers (PPH)
Explanations	How close is the meaning of the translation to the English word? There is no 100% matching translation to an English word, so literal translation is impossible in most cases, but we can strive to get the closest meaning in Chinese as much as possible.	Is this translation the most natural way to convey the meaning? The translation cannot be too formal or too vulgar, which will reduce the cultural trust, affinity, and acceptability of the translation.	Does the translation have strong negative connotations that would stigmatize dementia?
1	Yes	No	No
2	No	No	Yes
3	Yes	No	Yes
4	No	Yes	Yes
5	Yes	Yes	No
6	Yes	Yes	Yes

Revolving around the schematic diagram in Figure 1, we presented the results of this study in the following subsections.

### Mistranslations arising from the literal translation

We found the literal translation of Items 1, 2, 5, 9, and 16 problematic. Table 2 shows the specific literal translations and meanings of the literal translations of these items. It can be seen that the problems lay in the multiple meanings of the literal translation of “feel confident” in Item 1 and “touching” in Item 2, the possibilities of translating “supervise” in Item 5 and “ignore” in Item 16 into different Chinese phrases that have diverse meanings, and the lack of matching sayings in Chinese for “no longer themselves” in Item 9.

**Table 2:** Mistranslations of items 1, 2, 5, 9, and 16.

Problematic items	Original English phrasing	Literal translation	Meaning of the literal translation in Chinese culture
Item 1	Feel confident	Xìnxīn	A feeling of trust (in someone or something)
			A state of confident hopefulness that events will be favorable
			Any cognitive content held as true
			Belief in yourself and your abilities
			A strong belief in a supernatural power or powers that control human destiny
Item 2	Touching	Jiēchù	Deal with
			Close interaction
			Perceive via the tactile sense
			Come in contact with
			In physical contact
Item 5	Supervise	guǎnlǐ	The act of managing something—(neutral)
		kānguǎn	Keep tabs on, keep an eye on (slightly negative, informal language: adults to children)
		jiāndū	Watch and direct, oversee (moderately negative, formal language: authorities to individuals)
		jiānshì	Keep under surveillance, monitor (strongly negative)
Item 16	Ignore	hūshì	The trait of neglecting responsibilities and lacking concern
		mòshì	Willful lack of care and attention, disregard
		bù lǐcǎi	Fail to acknowledge, give little or no attention to
		qīngshì	Treat with contemptuous disregard
		lěngyù	A refusal to recognize someone you know
		mièshì	Look down on with disdain
		páichì	Marginalize, relegate to a lower or outer edge, as of specific groups of people
Item 9	No longer themselves	No matching sayings in Chinese	

### Root causes of mistranslations, implications for translation, and consequences of forced literal translations

The three factors identified as causes of the aforementioned mistranslations included: (1) The immediate Chinese direct translation can cause confusion because it has multiple Chinese meanings, (2) There is no immediate Chinese direct translation—multiple translations are possible with varying positive/negative emotions attached, and (3) There is the lack of counterpart culture-specific idioms in Chinese. In the final analysis, what underlay these three causes were three root causes, as listed in Table 3. Each of these root causes could provide an essential implication for translation, as shown in Table 3. Regardless of these implications, forced literal translations would incur severe consequences for the readers, as reported in Table 3. It follows that the forward-backward translation method proposed in previous studies (53–55, 60–62) did not work effectively in dementia stigma scale translation.

**Table 3:** Root causes of mistranslations, implications for translation, and consequences of forced literal translations.

	Root causes	Implications for translation	Consequences of forced literal translations
1	English and Chinese have different lexical systems.	One-to-one linear lexical matching is impossible since two large scenarios have been captured in our study: One English word was translated into one Chinese word with multiple meanings (See Questions 1 and 2) which could cause potential confusion. One English was translated to multiple competing words with distinct emotional and cultural connotations (see Questions 5 and 16) that could stigmatize dementia.	Misunderstanding and confusion to readers
2	Language registers (formality, abstractness) are different for health information in English and Chinese.	Adapting English formal expressions to more natural, informal Chinese words	Lowered cultural believability, trustworthiness, and communicative effectiveness to readers
3	Cultural expression repertoires in two cultures are distinct.	Using cultural equivalents in the target language to carry over the meaning (See Question 9, “people with dementia are no longer themselves”—changed to “changed into a different person”)	Meaningless translation to readers

### An assessment construct for person-centered translation of dementia public stigma scales proposed

**Figure 2:**
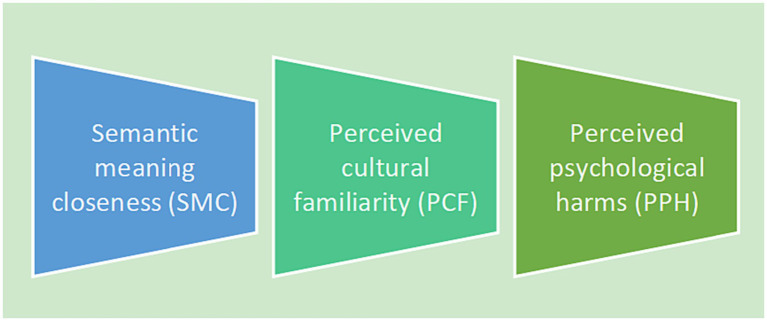
Assessment construct for the translation of dementia public stigma scales.

Based on the analysis above, we proposed a construct that could facilitate translating the DPSS into Chinese, as shown in Figure 2. This construct consists of three components: semantic meaning closeness (SMC), perceived cultural familiarity (PCF), and perceived psychological harms (PPH). It could be used as a model to guide the assessment of the Chinese translation of dementia stigma scales.

### Identification of the best translation among various translation options

Table 1 illustrates how our research team arrived at an agreed Chinese version of the DPSS before testing it for public use. A translated version was subjected to assessment in light of the three components comprising the construct shown in Figure 2. As can be seen from Table 1, we conducted six rounds of translation before finally agreeing on the best version of translation that satisfied these three components. During the repeated translating processes, we managed to achieve semantic meaning closeness to the English wordings of “feel confident” in Item 1 and “touching” in Item 2 by avoiding such possible literal translations as listed in Table 2 in rounds 1, 3, 5 and 6, as shown in Table 1. Similarly, we avoided using such Chinese phrases with diverse negative meanings listed in Table 2 when translating “supervise” in Item 5 and “ignore” in Item 16. Translating “supervised” and “ignore” into “bèi rén kānguǎnzhe” (watched over) and “Duǒ kāi” (avoid), respectively could ensure accuracy in the meaning that we conveyed through the translation and meanwhile possibly prevent perceived psychological harm to target readers in rounds 2, 3, 4, and 6, as shown in Table 1. As “are no longer themselves” in Item 9 has no corresponding culture-specific idioms in Chinese, we rendered it into a neutral wording of “Hǎoxiàng biànle yīgè rén” (appear to become another person) to achieve perceived cultural familiarity and acceptability to target readers, in rounds 4, 5, and 6, as shown in Table 1. In the six rounds of translation, only round 6 satisfies all three translation variants of SMC, PCF, and PPH. Therefore, the translation produced in round 6 was deemed as the best-translated version, that is, the final Chinese version of the DPSS, as shown in Supplementary Table S1.

## Discussion

We tentatively developed a method for person-centered translation of dementia public stigma scales in this study. The translator needs to discuss the meaning of the original text with someone with content knowledge to avoid misinterpretations and optimize word choice when there are multiple possible translations. To this end, we proposed an assessment construct for the translation of dementia public stigma scales that incorporates three major components: semantic meaning closeness (SMC), perceived cultural familiarity (PCF), and perceived psychological harms (PPH). Such a construct could help minimize mistranslations involved in the translation of dementia public stigma scales due to the differences in lexical systems, language registers, and cultural expression repertoires between the source and target languages. It can be used as a guide to help health translators navigate the translation of dementia public stigma scales. Translations following the method and the assessment construct we developed could facilitate understanding and measuring dementia public stigma.

We found that the forward and backward translation method did not work effectively in the translation of the DPSS into Chinese, detrimental to the understanding and measurement of dementia public stigma. Chang et al. (50), Zhao et al. (51), Mohamad et al. (52), Maneesriwongul and Dixon (53), Shan et al. (48), Sperber (55), Guillemin et al. (60), Sousa and Rojjanasrirat (61), and Sidani et al. (62), among many others, adopted forward and backward translation to adapt the English versions of some health-related measures into different languages. Although they concluded that this method was effective in their studies, we found it insufficient in our study. English and Chinese have different lexical systems, language registers, and cultural expression repertoires, which challenged the English-to-Chinese translation of the DPSS. These differences made it extremely difficult to forward-translate this scale into Chinese. For example, if “ignore” in Item 16 were forward-translated into “hūshì” (the trait of neglecting responsibilities and lacking concern), “mòshì” (willful lack of care and attention, disregard), “bù lǐcǎi” (fail to acknowledge, give little or no attention to), “qīngshì” (treat with contemptuous disregard), “lěngyù” (a refusal to recognize someone you know), “mièshì” (look down on with disdain), or “páichì” (marginalize, relegated to a lower or outer edge, as of specific groups of people), different degrees of discrimination or negative emotions would be induced, which is not intended in the original English scale. These translations would naturally lead to misleading backward translations, making translation equivalence testing (54, 55) considerably challenging. Translations thus produced could not effectively explore the cognitive, emotional, and behavioral domains of stigma held by the general public, therefore failing to gain a better understanding of dementia public stigma. Besides, the DPSS was written in a dementia-friendly language, in response to the appeal of Alzheimer’s Australia (64) in Dementia Friendly Language Position Paper 4, which advocates that “Language is a powerful tool” and “The words we use can strongly influence how others treat or view people with dementia.” Considering this appeal, we believed that the forward and backward translation method would possibly distort the original meaning and intent of the DPSS, bringing additional stigma to individuals with dementia. As a result, such translated scales could not objectively solicit and measure public attitudes toward people with dementia. As “a true translation proceeds by the motions of understanding and sympathy” (65, p. 211), a health translator needs to keep “constantly examining the relationship between word and experience, i.e., signifier and signified” (63). To this end in our translation process, we attached great importance not merely to “the relationship between word and experience” to achieve linguistic appropriateness and cultural relevance from the perspective of health translators but also to the understanding of and sympathy for those with dementia from the perspectives of the DPSS author and dementia content experts. It can be said that our translation team played the role of “a powerful agent for cultural change,” and our translation functioned as “a bridge-building space between the source and the target” (66). As a result, the dementia pubic stigma scale translation in our study could ensure a translated scale that could effectively measure dementia public stigma and facilitate our understanding of such stigma. It is well-known and widely published that translation and back-translation often present challenges. As Brislin (67) has pointed out, back-translation may lead to three potential pitfalls. Specifically, the back-translated text may support equivalence between the source and target texts although problematic translation may exist, when (1) the forward-and back-translators share a set of rules for translating words or phrases that are not truly equivalent, (2) the back-translator can infer what is meant by a poorly translated target text and reproduce the source text, or (3) the forward-translator retains the grammatical structure of the source text in the target text, therefore making it easy to back-translate while making it incomprehensible or awkward to monolingual target language speakers (67, 68). Therefore, it is crucial to rely on a team-based process, as described in this manuscript.

We proposed a better alternative, a method for person-centered translation of dementia public stigma scales, to reveal and measure such stigma more objectively. This method was effective in facilitating the translation of the DPSS in a culturally relevant and appropriate manner (54). It allowed us to use words friendly to people with dementia and their families, those that are “normal, inclusive, jargon-free, non-elitist, clear, straightforward, non-judgmental” (69), and those that center on the person rather than on the neurodegenerative disorder or the social care system (69). Such wording can avoid stripping individuals of their dignity and self-esteem, reinforcing inaccurate stereotypes, and heightening the fear and stigma surrounding dementia (64). Translated scales using such wording are most likely to assess stereotypes, prejudice, and discrimination among the general population, revealing their generalized negative beliefs, negative emotional reactions to stereotypes, and negative behavioral reactions resulting from prejudice (49). Our protocol can, therefore, be seen as an initiative counteracting the prevalent phenomenon that inappropriate language used in the literature, the media, and the community creates wrong descriptions, prescriptions, misconceptions, and stigma of individuals with dementia (69). A good case in point is such derogatory, stigmatizing, and discriminatory words as “demented,” “sufferers,” “subjects,” and “victims” used by most researchers and presenters at the 2014 Alzheimer’s disease International Conference (69). In the context that the language being used remains stigmatizing, negative, and disempowering (70), there is a pressing need to use “inclusive non-offensive language that supports the whole person positively, rather than negative demeaning language that stigmatizes and separates us” (69). In this case, the protocol we proposed in this study can contribute to the promoted use of person-centered, dementia-friendly language, especially in the translation of dementia public stigma scales. Counteracting inaccurate stereotypes and the resulting prejudice and discrimination against dementia, translated scales using such language could help us understand and assess the public attitudes toward dementia more objectively.

Our study also points to the need to construct a person-centered theory of translation (63) of dementia-related materials or in health care and medical domains in general. To this end, health translation studies should be taken away from purely linguistic and cultural analysis. Health translation in specific social and cultural circumstances needs to fulfill its expected social and cultural roles. As such, before engaging in translating health materials and constructing health translation theories, health translators and translation theorists should ask themselves the following question: “In whose terms, to which linguistic constituency, and in the name of what kind of intellectual authority does one translate?” (71). To answer this question, health translators and translation theorists need to adopt a person-centered approach advocated by Robinson (72) and Hoffman (65) to consider “what people need, what people can do and what people think and feel” (63). In the context of the prevalent social stigma attached to dementia, health translators and translation theorists need to spare no efforts to center on people with dementia and their relatives in their translation practices and theory construction to “change views of and about people with dementia,” “include them in the research and conversations about them” (63), and “remove the stigma which we hear of every day in dementia” (63). The language being used about individuals with dementia is a powerful tool (73) for inclusion, reducing stigma, and increasing education and awareness as the way forward in reducing stigma (74). Provided that a people-centered theory of translation in health care and medical domains can be established, the disadvantaged position of patients could be improved through dementia-friendly, inclusive, non-offensive language in the translated materials about dementia to some extent. Such a translation theory is “true to life” (63). Such translation theories are urgently needed, especially when considering that “Language creates the particularly human kind of rapport, of being together, that we are in a conversation together” (75).

To establish a person-centered theory of dementia translation, we need to highlight the importance of the translator’s role, which has already been stressed by famous translation scholars such as Bassnett (66), Robinson (72), Venuti (76), and Snell-Hornby (77). To be qualified in health and especially dementia translation, translators should be equipped with essential “literacies,” which include the ability to understand “what people need, what people can do and what people think and feel” (63), in addition to bilingual and bicultural competences (77). They also need to enhance translatability by focusing on practice and cognition (63) to make dementia translation “a humanizing process” (72).

### Strengths and limitations

To develop a method and an assessment construct for person-centered translation of dementia public stigma scales, we formed a research team comprising bilingual health educators, bilingual translators, the scale author, and content experts. Such a composition could ensure the quality of translation from different perspectives of experts in relevant domains, especially considering the interdisciplinary nature of dementia translation. Another strength lay in the bilingual translators’ experience in community-based health translation for many years. Their rich health translation practice could enable them to gain a keen, sensitive sense of cross-cultural and-lingual differences both from the perspective of language and from the perspective of health care. This is beneficial to ascertaining the key steps of the person-centered translation method and the core elements of the translation quality assessment construct we tried to develop. The translation method and the assessment construct we developed may be used as a guide to help navigate the translations of dementia public stigma scales that can be used to develop and evaluate interventions aimed at dementia public stigma reduction in the public.

To our knowledge, they are the first method and the first assessment construct for person-centered translation of dementia public stigma scales that have been developed. Without relevant studies for reference, our translation method and assessment construct may not be perfect. Their reliability and efficacy need to be validated in future studies. Their applicability to other dementia-related materials than dementia public stigma scales needs to be further attested. As stigma is a complex social construct and the DPSS was developed in English-speaking populations in Australia, the Chinese version of the DPSS we developed may not be perfectly specific to the Chinese language and culture although we made great efforts to adapt it linguistically and culturally. In the following stage of research, we would conduct a pretest (pilot study) to obtain initial psychometric results for the Chinese DPSS. During this process, participants would be invited to comment on the wording and comprehensibility of the question items to identify potential issues in the Chinese DPSS. Based on the findings from the pilot study, we would make adjustments to obtain the final Chinese version of the DPSS.

### Conclusion

The translation method and the assessment construct we developed are designed to facilitate the person-centered translation of dementia public stigma scales. They can help health translators navigate dementia translation to destigmatize people with dementia and their relatives while maintaining the original meaning and intent of the source text in a culturally relevant and appropriate manner in the target text. The best Chinese version of the DPSS we translated could be used for further evaluation with the public to test its psychometric properties. The translation method and the assessment construct we developed could be further validated for their reliability and efficacy in dementia public stigma scale translation and dementia translation in general.

## Data availability statement

The original contributions presented in the study are included in the article/Supplementary material, further inquiries can be directed to the corresponding author.

## Author contributions

JL, YS, and MJ: supervision, conceptualization, and methodology. MJ and WC: scale translation. YS and JL: writing—original draft, review, and editing, investigation, and formal analysis. YS and MJ: formal analysis, data curation, visualization, and project administration. JL, L-FL, SK, AB-W, and SS: critical review and commentary. JL: funding acquisition. All authors contributed to the article and approved the submitted version.
