# Supplementary material for: Development of a method and an assessment construct for person-centered translation of dementia public stigma scales
Source: Front Public Health. 2024 Jan 23;11:1233400. doi: 10.3389/fpubh.2023.1233400 (PMC10846308; doi:10.3389/fpubh.2023.1233400)
Supplement: Supplementary file 1 [file Table_1.DOCX]

**Table 4** The final Chinese version of the DPSS.

| **Question Items** | 1^a^ | 2 | 3 | 4 | 5 | 6 | 7 |
| --- | --- | --- | --- | --- | --- | --- | --- |
| Q1 我知道在得了痴呆症的人身边应该怎么做。 | 🌕 | 🌕 | 🌕 | 🌕 | 🌕 | 🌕 | 🌕 |
| Q2 当触碰得了痴呆症的人，我不会感到任何不适。 | 🌕 | 🌕 | 🌕 | 🌕 | 🌕 | 🌕 | 🌕 |
| Q3 在得了痴呆症的人的身边，我还是感到一样轻松、自然。 | 🌕 | 🌕 | 🌕 | 🌕 | 🌕 | 🌕 | 🌕 |
| Q4 我害怕那些得了痴呆症的人。 | 🌕 | 🌕 | 🌕 | 🌕 | 🌕 | 🌕 | 🌕 |
| Q5 得了痴呆症的人应该总是被人看管着。 | 🌕 | 🌕 | 🌕 | 🌕 | 🌕 | 🌕 | 🌕 |
| Q6 得了痴呆症的人行为和言语都不好预测。 | 🌕 | 🌕 | 🌕 | 🌕 | 🌕 | 🌕 | 🌕 |
| Q7 得了痴呆症的人很像孩子一样。 | 🌕 | 🌕 | 🌕 | 🌕 | 🌕 | 🌕 | 🌕 |
| Q8得了痴呆症的人无法做任何个人决定。 | 🌕 | 🌕 | 🌕 | 🌕 | 🌕 | 🌕 | 🌕 |
| Q9得了痴呆症的人得了这个病之后就像变了一个人。 | 🌕 | 🌕 | 🌕 | 🌕 | 🌕 | 🌕 | 🌕 |
| Q10 得了痴呆症的人也可以享受生活. | 🌕 | 🌕 | 🌕 | 🌕 | 🌕 | 🌕 | 🌕 |
| Q11得了痴呆症的人也能够感受到别人对他们的关爱。 | 🌕 | 🌕 | 🌕 | 🌕 | 🌕 | 🌕 | 🌕 |
| Q12 和得了痴呆症的人很好地互动，这也是有可能的。 | 🌕 | 🌕 | 🌕 | 🌕 | 🌕 | 🌕 | 🌕 |
| Q13得了痴呆症的人对他们的家人来说是一种负担。 | 🌕 | 🌕 | 🌕 | 🌕 | 🌕 | 🌕 | 🌕 |
| Q14得了痴呆症的人对医疗系统来说是一种负担。 | 🌕 | 🌕 | 🌕 | 🌕 | 🌕 | 🌕 | 🌕 |
| Q15我不会让得了痴呆症的人参加各种活动。 | 🌕 | 🌕 | 🌕 | 🌕 | 🌕 | 🌕 | 🌕 |
| Q16 我会躲开得了痴呆症的人。 | 🌕 | 🌕 | 🌕 | 🌕 | 🌕 | 🌕 | 🌕 |

^a^ 1 = Strongly Disagree; 2 = Disagree; 3 = moderately disagree; 4 = not disagree nor agree; 5 = moderately agree, 6 = agree; 7 = strongly agree.
